# Supplementary figures and images for: Rothia nasimurium as a Cause of Disease: First Isolation from Farmed Chickens
Source: Vet Sci. 2022 Nov 22;9(12):653. doi: 10.3390/vetsci9120653 (PMC9783258; doi:10.3390/vetsci9120653)

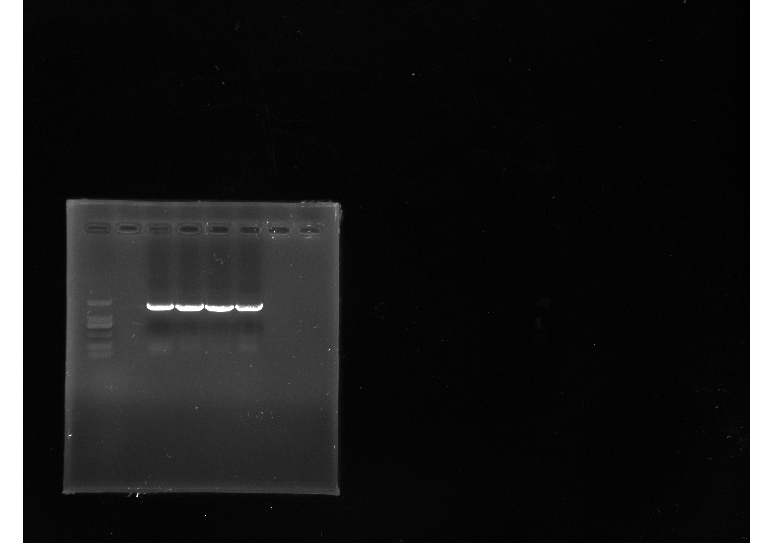

Supplement: Supplementary file 1 [file vetsci-09-00653-s001.zip › Figure S1 The original western blot of Figure 3.tif]

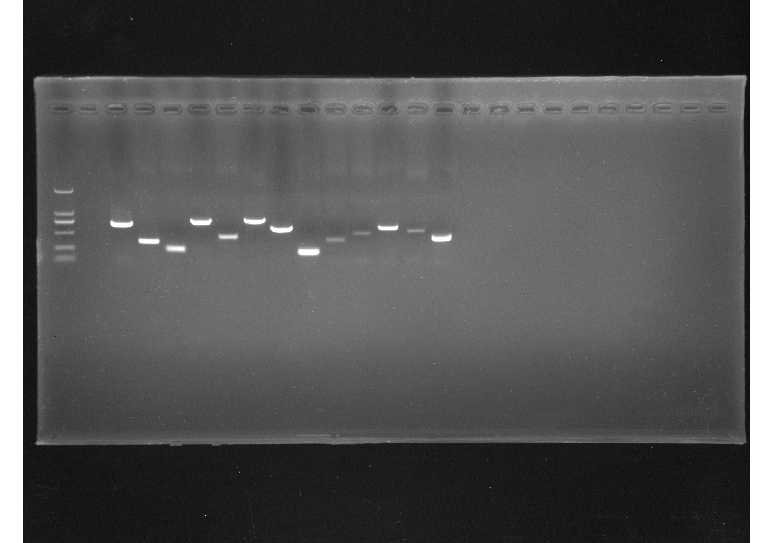

Supplement: Supplementary file 1 [file vetsci-09-00653-s001.zip › Figure S2 The original western blot of Figure 5.jpg]
